# Coronoid process sclerosis as a reproducible and highly heritable risk predictor of medial coronoid disease in labrador retrievers

**DOI:** 10.3389/fvets.2026.1761592

**Published:** 2026-02-09

**Authors:** Birgit Deboutte, Emmelie Stock, Kaatje Kromhout, Annika van der Wal, Katrien Vanderperren, Bart J. G. Broeckx

**Affiliations:** 1 Department of Veterinary and Biosciences, Faculty of Veterinary Medicine, Ghent University, Ghent, Belgium; 2 Department of Veterinary Morphology, Imaging, Orthopedics, Rehabilitation and Nutrition, Faculty of Veterinary Medicine, Ghent University, Ghent, Belgium; 3 Hulphond Nederland, Herpen, Netherlands

**Keywords:** assistance dogs, computed tomography, coronoid process sclerosis, elbow dysplasia, heritability, labrador retriever, medial coronoid disease, selective breeding

## Abstract

**Introduction:**

Canine elbow dysplasia (CED) is an important cause of exclusion during training for assistance dogs, making improved selection strategies essential for these programs. Although selective breeding has been applied for decades, progress in reducing CED prevalence has been limited, partly because many breeding programs rely on radiography rather than the more accurate computed tomography (CT). This study aimed to evaluate CT-based scoring of specific features in breeding dogs, as a tool to reduce the prevalence of medial coronoid disease (MCD)-the most common manifestation of CED-in their offspring.

**Methods:**

An observational study was conducted on a group of Labrador Retrievers from two assistance dog breeding programs where CT is used routinely for screening. Six CT features were scored in parents without primary lesions, and their association with MCD occurrence in offspring was analyzed. Inter-rater agreement was assessed, and for features deemed reproducible, CT reports from a subset of offspring were rescored and narrow-sense heritability was estimated using Bayesian statistical methods.

**Results:**

Sclerosis of the coronoid process (CP) was the strongest predictor of offspring MCD risk [odds ratio = 4.6, 95% CI = (1.3; 16.5)], had sufficiently high inter-rater agreement and showed high heritability.

**Discussion:**

CP sclerosis proved to be a significant and reproducible phenotype with high heritability, encouraging the use of this feature in CT-based scoring protocols in breeding programs to improve selection strategies and accelerate progress in reducing MCD prevalence.

## 1 Introduction

Despite years of research and screening efforts, canine elbow dysplasia (CED) remains a challenge for many veterinary clinicians and dog breeders. The condition also has a considerable impact on working dog populations. In assistance dogs, CED is one of the major medical reasons for exclusion during training, indirectly contributing to the long waiting lists for these services (1). CED is not a single condition, but rather an umbrella term covering several types of developmental abnormalities that lead to osteoarthritis of the elbow joint: medial coronoid disease (MCD), ununited anconeal process (UAP), osteochondritis dissecans (OCD) of the medial humeral condyle and elbow incongruity (2–4). Osteoarthritis arises from cartilage damage, medial joint instability and ongoing irritation of the affected elbow joint (5, 6). Among these subtypes, MCD is the most frequently diagnosed and one of the most common causes of forelimb lameness in large and giant dog breeds (3, 7–9).

Accurate detection of CED is challenging; lameness is not always observed, and radiographs often fail to reveal early or subtle cases (4, 9–11). In contrast, computed tomography (CT) provides superior sensitivity and specificity for identifying abnormalities across all CED subtypes (4, 11–14).

CED is a multifactorial disease recognized to have a strong genetic component (15, 16). For Labrador Retrievers, heritability estimates for CED in general range between 0.1 and 0.19 (17–20) and phenotypic selection has led to disappointingly slow genetic progress, especially when compared with the improvements achieved in canine hip dysplasia (CHD) (19–23). Overall, in selective breeding programs aimed at reducing disease incidence, it is critical to base selection on phenotypes that are reproducible, have a high heritability and can be measured with a high sensitivity and specificity (4, 24). In that context, studies investigating the link between radiographic findings in parents and progeny can provide additional insights.

Specifically in the context of CED, there is still merit in evaluating the effect of phenotyping protocols. In more detail, the IEWG classification was developed to classify dogs based on radiography, while CT-based screenings are becoming more common nowadays. The IEWG considers sclerosis of the coronoid process as a mild to moderate manifestation of elbow dysplasia, depending on the severity of the sclerosis (25), and MCD is frequently associated with sclerosis of the trochlear notch as well as radioulnar incongruity (9, 26, 27). However, no studies have directly investigated whether these radiographic changes in parents are associated with an increased risk of developing MCD in their offspring.

In this four-part stepwise observational study involving two Labrador Retriever populations, we first provided an overview of CED prevalence. We then assessed in the second part whether specific parental CT features were significantly predictive for MCD in their offspring in various subpopulations. Thirdly, we evaluated the interobserver agreement on those features that were significantly predictive. Finally, in the fourth part, we estimated the heritability of the significant and reproducible features associated with MCD.

## 2 Materials and methods

### 2.1 Study population

Data from two breeding programs for assistance dogs was used. One program is based in Belgium (population A), the other one is in the Netherlands (population B). In these populations, CT screening is routinely performed at approximately 12 months of age.

### 2.2 Study design: inclusion and exclusion criteria

In this four-part study, inclusion criteria gradually became more stringent. In the first part of the study, the focus was on describing the general CED prevalence, as well as the primary lesions (MCD, UAP, and OCD) to get an overview of CED characteristics. To be included in the first part of the study, an individual dog thus had to be CT-screened, and the screening report had to be available.

In the second part of the study, the goal was to identify CT features in parents that were predictive for MCD in their progeny as MCD was the most common primary CED finding observed in this population. CED cases other than MCD were thus excluded as the sample size was too low for a meaningful analysis (exclusion criterion). Overall, the group studied in this part was composed of:

- Parents with original CT images available for detailed,
- And their CT-screened offspring for which the screening report was available.

Three subgroups were defined based on the number of parents that were CT screened: both parent's CT images available (smallest group), only sire, and only dam. The population overall contained all individuals where at least one parent had CT images available and was thus the largest group. Significantly predictive was defined as “a significant odds ratio (OR) in the subgroup where information was available from both parents.”

In the third part, the goal was to evaluate the interobserver agreement of the CT features that were scored. Practically, the group studied in this part was thus the parents for which the CT images were available (inclusion criterion).

**Figure 1:**
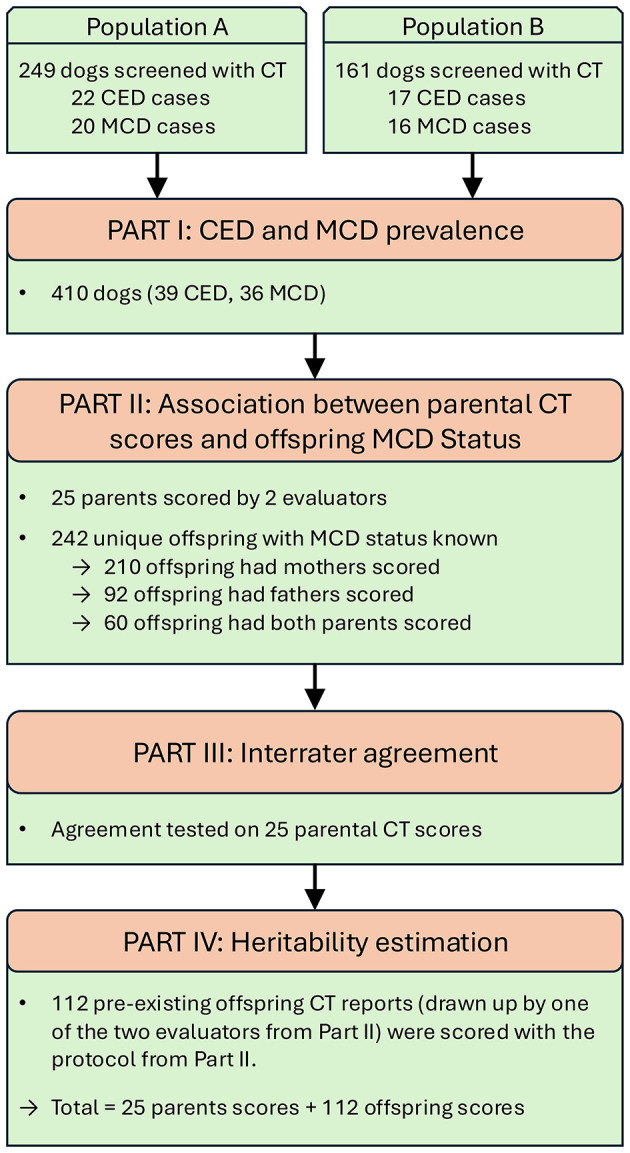
Overview of the sample distribution, including the number of dogs, parents, offspring, and datasets used across the four analyses. CED, canine elbow dysplasia; MCD, medial coronoid disease; CT, computed tomography.

In the fourth part, the goal was to estimate the heritability of those CT features that were associated with MCD and were sufficiently reproducible. CT-screened offspring in which the reports followed the CT evaluation protocol as described below, were retained, as well as the parents that were scored according to this protocol. An overview of the sample distribution, including the number of dogs, parents, offspring, and datasets used across the four analyses, is provided in Figure 1.

### 2.3 CED evaluation

CED evaluations were based on the original screening reports (part one), scores obtained with the specific CT screening protocol described below (part three) or a combination (part two and four).

### 2.4 Report evaluation

For part one, the presence of primary lesions (MCD, UAP, and OCD) was recorded, based on an evaluation of the available CT screening reports. For part two, the presence of primary lesions (MCD, UAP, and OCD) of the progeny was also based on these CT screening reports, while parental features were scored as described below. For part four, the CT screening reports of the progeny, evaluated by one of the two observers that were shown to score reproducible in part three, and that followed the CT evaluation protocol described below, were used.

### 2.5 CT evaluation protocol

The CT scans from the Dutch assistance-dog breeding program were acquired using a Siemens Somatom Definition AS CT system (Siemens Healthineers, Erlangen, Germany) with a helical modality. Dogs were scanned under sedation in dorsal recumbency with the thoracic limbs extended cranially and the elbows positioned at 90° of flexion to ensure consistent and unobstructed visualization. Both elbows were scanned at the same time. Images were obtained at 140 kVp and 125 mAs, and a 1.0 mm slice thickness. CT scans from the Belgian assistance-dog breeding program were acquired using a 320-row MDCT unit (Aquilion One, Toshiba Medical Systems, Otawara, Japan). Positioning was identical to the Dutch protocol, with dogs placed in dorsal recumbency with the elbows extended cranially, allowing simultaneous imaging of both elbows. A helical acquisition modality was used, with 120 kVp, 150 mAs, and a 0.5 mm slice thickness. The DICOM files were retrieved, and each series was reviewed using OsiriX version 14.1 DICOM viewer.

**Figure 2:**
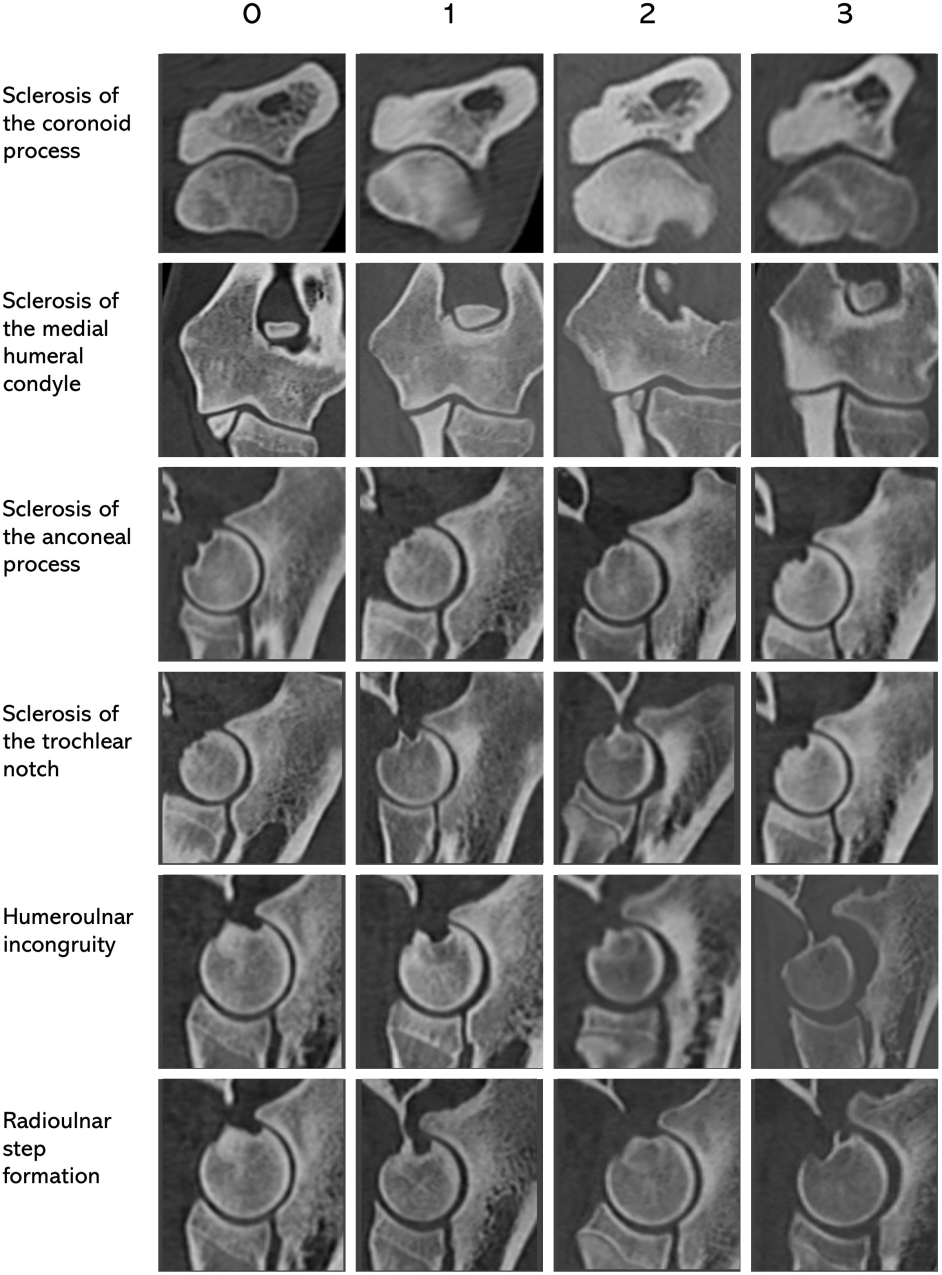
CT images of canine elbows showing the scoring protocol for six diagnostic characteristics used in the evaluation of elbow dysplasia. 0 = absent; 1 = mild; 2 = moderate, 3 = severe.

Aside from the presence of primary lesions (i.e., MCD, UAP, and OCD), six specific characteristics of the elbow joint were scored on the CT scans, with a result recorded for each side, on a scale from 0 (absent) to 3 (severe): sclerosis of the coronoid process (CP), sclerosis of the medial humeral condyle (MHC), sclerosis of the trochlear notch (TN), sclerosis of the anconeal process (AP), humeroulnar incongruity and radioulnar step formation (Figure 2).

In part two, scoring was performed independently and blind by one board-certified veterinary radiologist (ES) and one experienced (>20 years of experience) veterinary radiologist (KK), both of which routinely evaluate CTs for CED screening. For part four, each time only one of the aforementioned evaluators did the scoring according to this protocol.

### 2.6 Statistical analysis

For part 1, descriptive statistics were used, detailing the number and proportion of CED cases overall and per primary lesion.

For part 2, the primary hypothesis was that parental scores for one or more features would be associated with increased odds of the offspring being diagnosed with MCD. Using a generalized linear mixed model (GLMM) with likelihood ratio testing, associations between each parental feature score and progeny MCD (yes/no) diagnosis were evaluated for progeny for which both parents scores were available. Parent identity was added as a random effect, to model non-independence due to shared parentage across/within litters. Features were retained if they were statistically significant (*P* < 0.05). These variables were then tested for the progeny with maternal scores, progeny with paternal scores and all progeny together, where at least one parent was scored, using the same GLMM structure. As scores of two observers were available, these were averaged here to reduce the influence of individual observers.

For part three, interrater reliability for the significant MCD-related features (identified in the previous analysis) was assessed using squared weighted Cohen's kappa (28). Only features with moderate agreement (κ ≥ 0.4) (29) were considered acceptable. For this part, the original scores of both evaluators were used.

In part 4, the narrow-sense heritability (*h*^2^) of those features where interrater agreement was sufficiently high for it to be scored by one observer (30), was estimated using Bayesian mixed models implemented in the “MCMCglmm” package in R (31), following (32), based on the subset of offspring and parents as described earlier. Given the semi-quantitative and ordinal nature of the CT scores, heritability was evaluated under two modeling frameworks. First, a linear animal model was fitted, treating the scores as approximately continuous, given that the observed score distribution was unimodal and approximately symmetric, yielding a *h*^2^ estimate on the observed scale. Second, a threshold animal model was fitted, given the ordinal nature of the scores and assuming an underlying continuous liability, to estimate *h*^2^ on the liability scale. In both models, additive genetic effects were modeled using the pedigree-based animal term, and a litter random effect was included to account for common early environment. A likelihood ratio test indicated that sex nor side of the elbow were associated with the scored feature, so neither was included as a fixed effect. For the linear model, the starting value for *V*_*e*_ (residual variance) was set to half of the total phenotypic variance and for *V*_*a*_ (additive genetic variance) and *V*_*l*_ (litter variance) they were both set to 0.25 of the total phenotypic variance. For the threshold model, *V*_*e*_ was fixed at one and starting values for *V*_*a*_ and *V*_*l*_ were also set to one. Sensitivity of posterior heritability estimates to prior specification was assessed by refitting both linear and threshold models under multiple inverse-Wishart priors for variance components, ranging from weak (nu = 0.002) to stronger (nu = 2) prior assumptions. Posterior convergence was assessed using trace plots and autocorrelation diagnostics. The h^2^ was estimated as the mean of the posterior distribution of the proportion of additive genetic variance relative to total phenotypic variance. To ensure the models were not biased toward unrealistically high heritability, we also ran a negative-control analysis using a randomly generated phenotype with no genetic basis. The heritability estimate converged to zero for both model types, confirming appropriate model behavior.

## 3 Results

### 3.1 Population characteristics

A total of 410 Labrador Retrievers were included in this study, originating from two breeding programs for assistance dogs, one based in Belgium (population A, *n* = 249) and the other in the Netherlands (population B, *n* = 161). Four males from population B had offspring in population A, and one male from population A had offspring in population B.

### 3.2 Part 1: CED prevalence

Of the 410 dogs included in the first part of the study, the CED prevalence was 9.5 % (*n* = 39), of which 34 had MCD, two had both MCD and OCD, one had OCD and two had osteoarthritis without primary lesions. None had UAP.

### 3.3 Part 2: parental phenotypes and their influence on MCD

CT images of 25 parents were scored by the two evaluators, i.e., results for 50 elbows were available. These were all free from MCD, UAP, OCD and osteoarthritis. In total, these parents had 242 offspring from 40 litters with at least one parent scored, there were 60 offspring with both parents scored, 92 with paternal scores and 210 with maternal scores, respectively. Of the parents with scored CT images, 15 were dams and 10 were sires; among the dams, five produced 4 litters, one produced 3 litters, three produced 2 litters, and six produced 1 litter, while among the sires, two produced 3 litters, one produced 2 litters, and the remaining eight produced 1 litter each.

**Figure 3:**
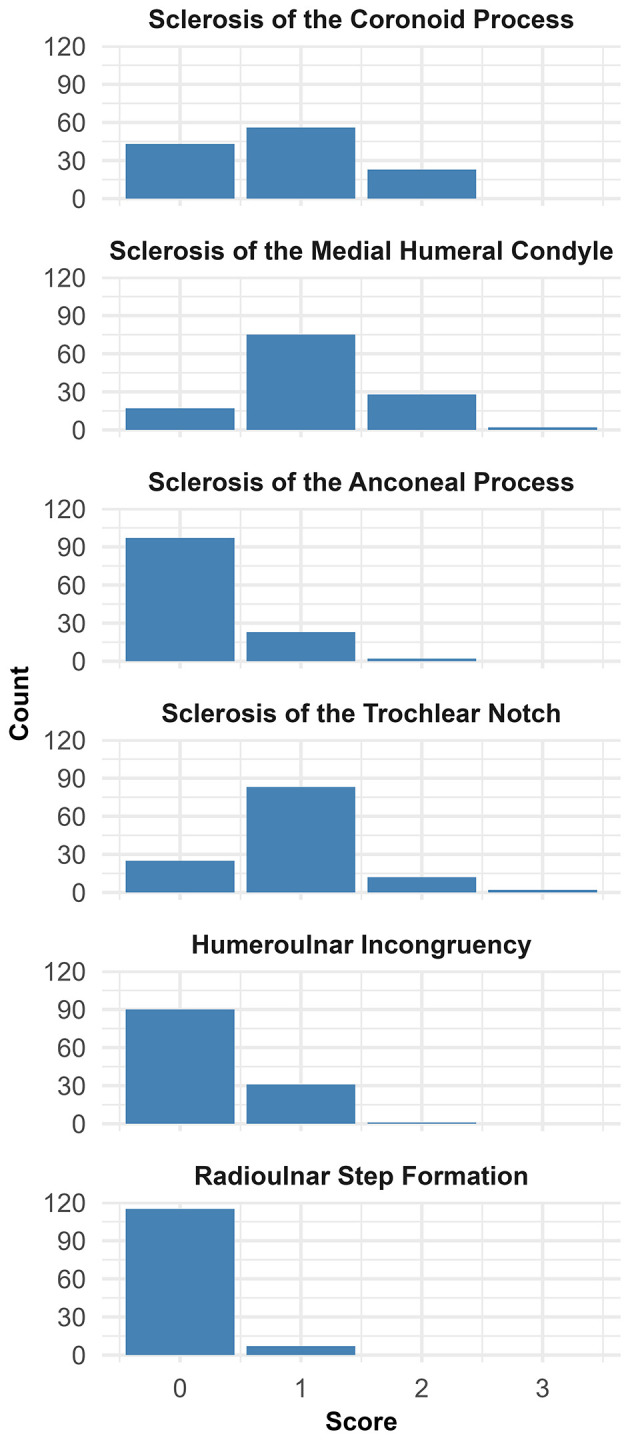
Graphical representation of the distribution of the scores for each of the six CT characteristics.

In these parents, the median scores for sclerosis were 1 (range = 0–3) for the medial humeral condyle, 1 (range = 0–2) for the coronoid process, 1 (range = 0–3) for the trochlear notch, and 0 (range = 0–2) for the anconeal process. For the incongruity-related features, humeroulnar incongruity had a median score of 0 (range = 0–2), and radioulnar step formation had a median score of 0 (range = 0–1). The distribution of the 6 CT features is depicted graphically in Figure 3.

**Figure 4:**
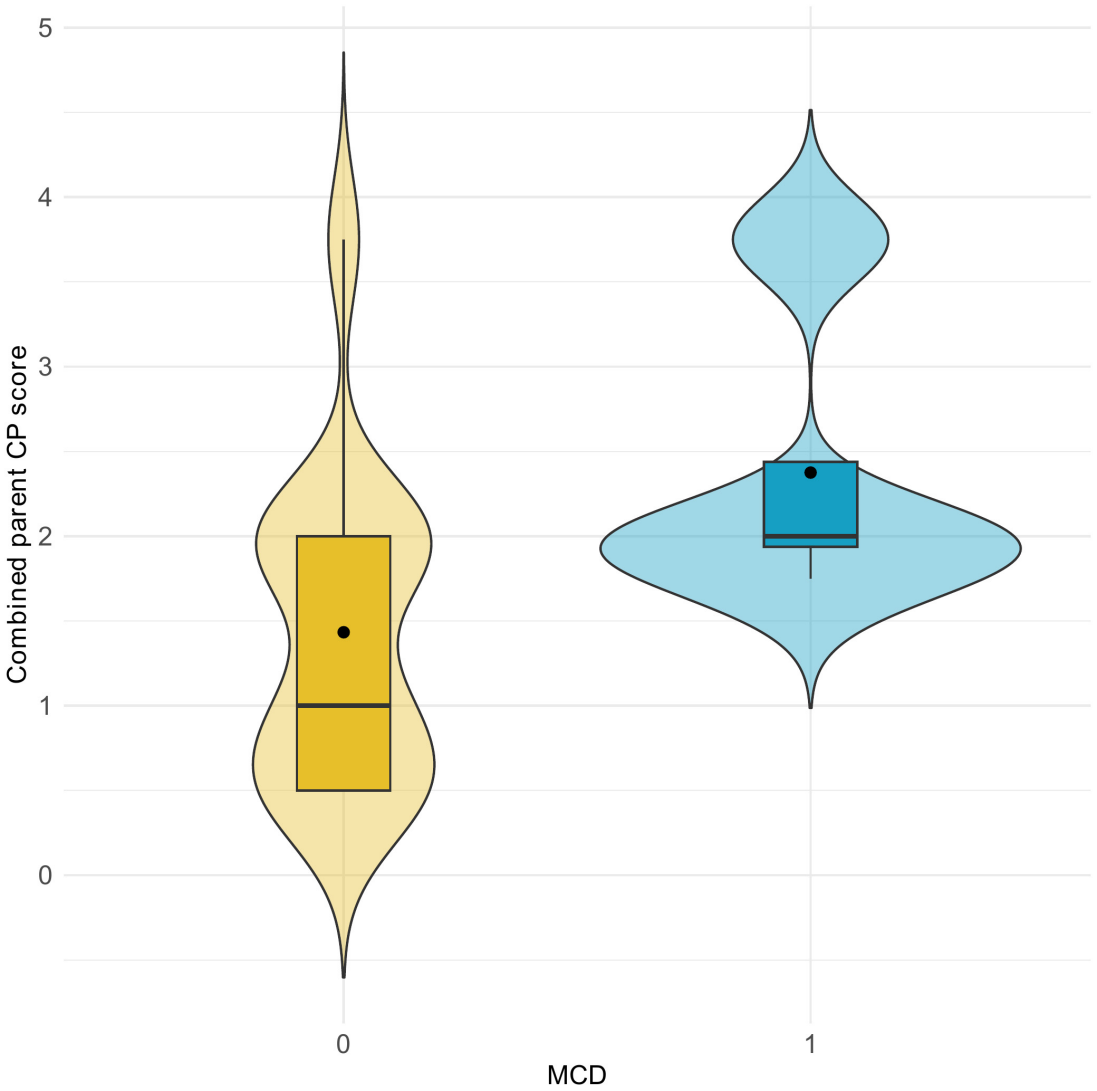
Violin plot of combined parental scores for sclerosis of the coronoid process (CP) in relation to the occurrence of medial coronoid disease (MCD) in offspring. 0 = unaffected; 1 = affected.

One features was shown to have a significant association with MCD diagnosis in the progeny with both parent scores available; a one-point increase in the score of sclerosis of the CP was associated with a more that four-fold increase in the odds of MCD in the offspring [OR = 4.6, 95% CI = (1.3; 16.5), *P* < 0.05], after adjusting for clustering by parent. Additionally, none of the offspring from parents with a combined score below 3 for sclerosis of the CP were diagnosed with MCD (Figure 4). Sclerosis of the CP showed consistent associations across maternal, paternal, and combined-parent analyses. The estimated effect was larger for the paternal scores [OR = 10.5, 95% CI = (1.2; 89.4), *P* < 0.05], while the maternal-only model showed a similar direction of effect but did not reach statistical significance [OR = 3.7, 95% CI = (0.9; 15), *P* = 0.063]. The combined model including all offspring with at least one parent scored confirmed the association [OR = 5.1, 95% CI = (1.5; 17.6), *P* < 0.05]. The effect of sclerosis of the MHC, the AP and the TN, humeroulnar incongruity and radioulnar step formation on the occurrence of MCD in the offspring was not statistically significant (Table 1).

**Table 1:** Odds ratios (OR) with 95% confidence intervals (CI) and *p*-values for the association between parental elbow CT scores and the occurrence of medial coronoid disease (MCD) in offspring, after adjusting for clustering by parent.

**Elbow CT image scores in parents**	**Both (*****n*** = **60)**			**Fathers (*****n*** = **92)**			**Mothers (*****n*** = **210)**			**All offspring (*****n*** = **242)**		
	**OR**	**95% CI**	***p*** **-value**	**OR**	**95% CI**	***p*** **-value**	**OR**	**95% CI**	***p*** **-value**	**OR**	**95% CI**	***p*** **-value**
Sclerosis of CP	4.6	[1.3; 16.5]	< 0.05	10.5	[1.2; 89.5]	< 0.05	3.7	[0.9; 15]	0.06	5.1	[1.5; 17.6]	< 0.05
Sclerosis of MHC	7.9	[0.8; 78.8]	0.08	–	–	–	–	–	–	–	–	–
Sclerosis of AP	0.5	[0; 12.5]	0.6	–	–	–	–	–	–	–	–	–
Sclerosis of TN	1.8	[0.2; 16.6]	0.6	–	–	–	–	–	–	–	–	–
Humeroulnar incongruity	0.7	[0; 19.5]	0.8	–	–	–	–	–	–	–	–	–
Radioulnar step formation	0.4	[0; 42.8]	0.7	–	–	–	–	–	–	–	–	–

Results are shown separately for offspring with both parents scored, fathers scored, mothers scored, and for all offspring combined with at least one parent scored. CP, coronoid process; MHC, medial humeral condyle; AP, anconeal process; TN, trochlear notch.

### 3.4 Part 3: inter-rater reliability

Inter-rater reliability was assessed for the significantly predictive parental CT-scored feature using squared weighted Cohen's kappa. Moderate inter-rater agreement (κ ≥ 0.4) was found for sclerosis of the coronoid process (κ = 0.44).

### 3.5 Part 4: heritability

**Figure 5:**
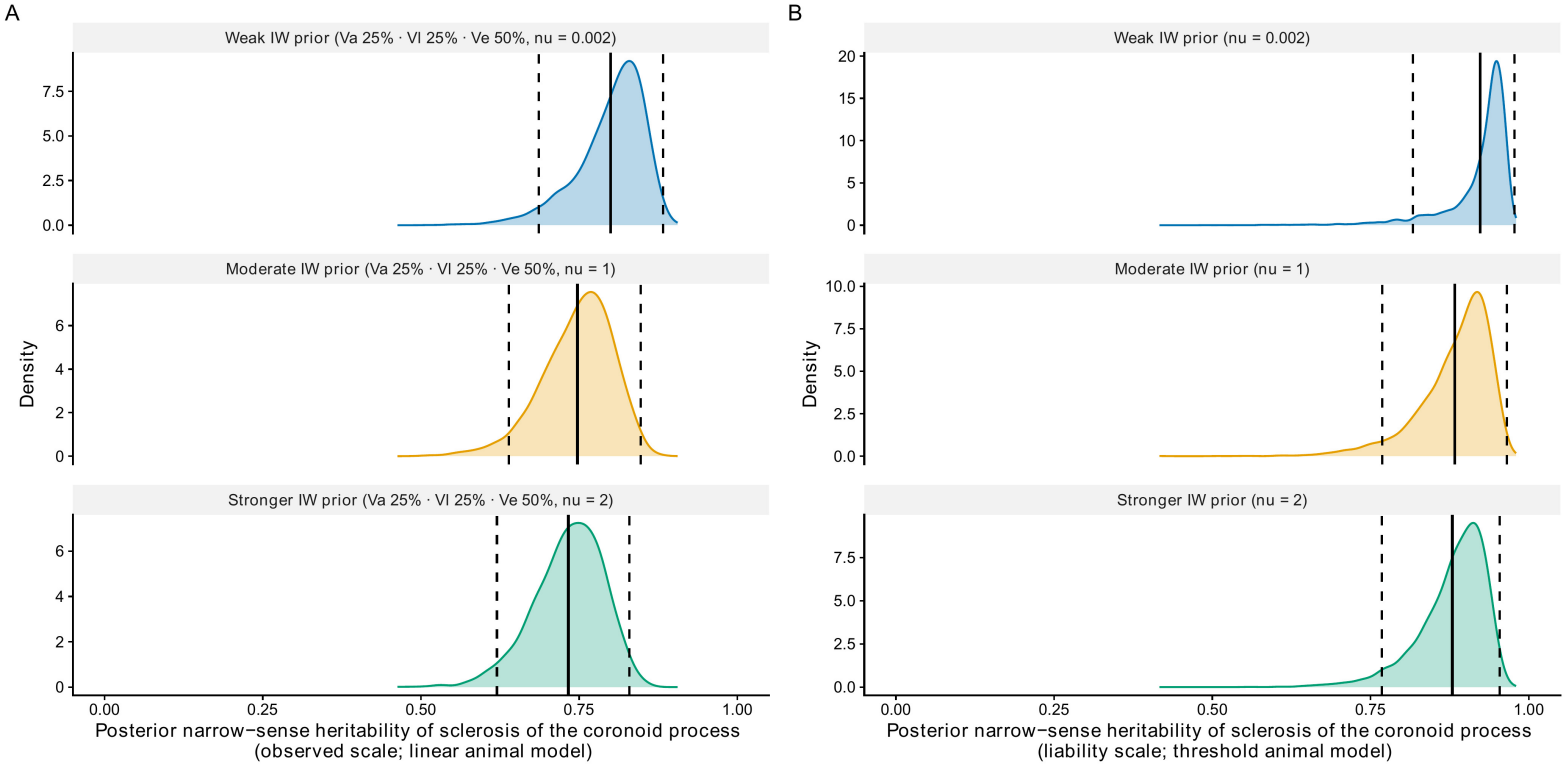
Posterior distributions of narrow-sense heritability (**h**^**2**^) of sclerosis of the coronoid process estimated using Bayesian linear **(A)** and threshold **(B)** animal models under weak, moderate, and stronger inverse-Wishart (IW) priors. Solid lines indicate posterior means and dashed lines indicate 95% highest posterior density intervals. **V**_**e**_ = residual variance; **V**_**a**_ = additive genetic variance; **V**_**l**_ = litter variance.

Sclerosis of the coronoid process was both significantly associated with MCD in offspring and met the κ ≥ 0.4 criterion and was retained for heritability estimation. Sclerosis of the CP was then scored from 112 screening reports available for dogs from the same population. When adding the scores of the 25 parents, this resulted in a total sample size of 137 animals available for *h*^2^ estimation. Neither sex (*P* = 0.71) nor elbow side (*P* = 1.00) had a significant effect on CP score and were not included in the animal models. Under the linear model, observed-scale *h*^2^ estimates for sclerosis of the coronoid process were high, with posterior means ranging from 0.73 to 0.80 across different prior specifications and 95% highest posterior density intervals (95% HDI) spanning 0.62–0.89. Under the threshold model, heritability on the liability scale was even higher, with posterior mean *h*^2^ estimates ranging from 0.88 to 0.92 and 95% HDI spanning 0.77–0.98 (Figure 5). MCMC diagnostics confirmed adequate convergence for all models.

## 4 Discussion

Orthopedic diseases like CED and CHD are among the most predominant reasons for rejections of assistance dogs in training (1). Recently, a study conducted by our group found that CHD prevalence can be significantly reduced by combining the standard ventrodorsal hip-extended radiograph with a stress radiograph focusing on quantifying hip joint laxity (22). Similarly, the implementation of CT as a routine screening method aimed to achieve the same goal. Yet, as illustrated in the first part of this study, even when parents are free of primary lesions (i.e., MCD, OCD, UAP as defined by the IEWG), still a 9.5% CED prevalence was found. As such, the aim was to search for other CT features in parental CT scans that seem to be associated with CED in their progeny.

It is no surprise that the current study was predominantly focused on MCD. Overall, it has been reported as the most frequent of the primary lesions in CED (7) and also here, it accounted for 92.3 percent of the CED (36/39) cases. Practically, this also means that strategies that successfully allow elimination of this part of the CED complex, would more or less eradicate close to all CED-linked rejections in the assistance dog population studied.

In a search for more subtle features that might be associated with MCD, the relatively limited variability of humeroulnar incongruency, as well as radioulnar step in the current study population, is noticeable. This can be explained by several factors. First, the CT images assessed were from animals selected for breeding, meaning they had likely been chosen precisely because they showed a favorable elbow conformation. Second, the Labrador Retriever populations included in this study were bred within two assistance dog breeding programs, where CED screening is more rigorous than in the general population, which should result in healthier elbows overall. Irrespective of the cause, the limited variability made it less likely to identify effects for the radioulnar step and humeroulnar congruency. As a consequence, the study population is not fully representative of the general pet population in terms of score distribution and variability. In addition, the number of parental CT scans available for this study was limited, restricting statistical power, particularly for less prevalent features. Especially for applicability to the general pet population, future studies should thus include parents with a wider range of scores from a more diverse population with a broader genetic and phenotypic background, while irrespective of the target population, a larger number of parental CT images would be beneficial.

Nevertheless, increased sclerosis of the CP was associated with a higher risk, with the odds of MCD in the offspring increasing approximately more than four-fold for each one-point increase in score when both parent CT scores were available, even after adjusting for clustering by parent. The association between parental CP sclerosis and offspring MCD was consistent across analyses of maternal, paternal, and combined parent scores. While the association reached statistical significance in the paternal and combined models, the maternal-only model also showed a directionally consistent effect, but with borderline significance (*P* = 0.06). This pattern suggests a true underlying effect that may have failed to reach significance in the maternal subset due to increased variability or reduced statistical power.

While these findings point to a clear association between parental CP sclerosis and offspring MCD risk, several aspects of the study impose boundaries within which the results should be interpreted. The limited variability observed for humeroulnar incongruity and radioulnar step in this population likely reflects selection of breeding animals with favorable elbow conformation, as well as the rigorous screening practices within assistance dog breeding programs. The ongoing selection in the assistance dogs populations might have reduced the power to detect effects for features that may still play an important role in the general population.

The strong association between sclerosis of the CP in the parents' elbows and the risk of MCD in their offspring was anticipated (5, 25, 27), and the scoring system used here enabled a more precise quantification of this effect. The IEWG grading scheme could be considered to serve a similar goal, as sclerosis of the CP is among the criteria included. However, since the IEWG system grades CED in its entirety, the present scoring system may provide a framework more specifically adapted to MCD. None of the offspring in this sample developed MCD when the combined parents score was below 3 for CP sclerosis, which suggests that informed combinations with respect to this feature may effectively limit the overall MCD risk. Importantly, this can be achieved while maintaining genetic diversity, as it minimizes the need to exclude animals from the breeding pool. In fact, all 25 parents included in this study could have been paired in ways that kept their combined score below this threshold. It should be noted that the proposed scoring threshold and predictive value were derived from a single cohort and would need to be validated in independent populations before broader implementation.

When ORs are compared across various parental groups for sclerosis of the CP, the effects appeared greater for fathers than for mothers, with the maternal results just failing to reach significance. This should be interpreted with caution, however, as fewer paternal scores were available compared to maternal scores (maternal scores were available for 210 offspring, with 20 MCD cases, and paternal scores for 92 offspring, with 5 MCD cases), which can affect estimates. These apparent differences are therefore more likely due to data structure than true sex-specific effects. Robust inference about differences between maternal and paternal scores would require a more balanced dataset or models explicitly accounting for unequal parent representation. Still, the estimated effect for CP sclerosis remained notable even when only one parent was included, suggesting that scoring from just one parent can already provide insights to reduce MCD prevalence.

Sclerosis of the CP had a Cohen's squared weighted kappa value above 0.40, indicating at least moderate agreement between the two raters. This implies that another box is ticked of what a feature ideally has in terms of characteristics to base screening on. A reproducible scoring protocol makes implementation easier and less prone to personal interpretation. To aid implementation also outside this study, we provided a figure that can be used as a visual guide to base the classification on (Figure 3). Nevertheless, the moderate level of agreement indicates that some degree of subjectivity remains, and further evaluation and refinement of the scoring protocol will help to reduce residual subjectivity and measurement error.

Aside from being the most logical, consistent predictor of MCD risk in the progeny, sclerosis of the CP also had a high heritability estimate, which further supports its value as a characteristic on which to base breeding decisions. Practically, a high *h*^2^ implies a higher predictability of progeny scores based on parental values, but also that the response to selection will be high. A gradual reduction of parental CP scores will thus likely reduce CP sclerosis and overall MCD susceptibility in future generations.

This study focused on heritability and phenotypic associations, and did not incorporate molecular genetic data. As such, the underlying molecular genetic mechanisms associated with MCD remain unresolved. To date, no genetic loci or molecular markers have been specifically associated with MCD, and most genomic studies have treated CED as a single phenotype (33–35). However, as CED is more an umbrella term for several different developmental abnormalities (i.e., MCD, UAP and OCD), we suggest future genetic and genomic studies separate CED into its specific subtypes. This could allow for more precise identification of associated genetic risk factors, relevant to each respective subtype.

In conclusion, the use of CT imaging ensuring high accuracy, the moderate interrater agreement suggesting acceptable reproducibility and the high heritability indicating a strong genetic basis, this study demonstrates that the proposed scoring protocol may provide a robust phenotypic basis that could be integrated into breeding strategies aimed at reducing the risk of MCD. Thus, while CT might represent a higher initial investment compared to radiography, its use within specialized breeding programs may offer considerable long-term benefits, particularly within assistance dog breeding programs where it may help reduce training losses. Future studies including larger and more phenotypically diverse populations will be valuable for refining thresholds and validating predictive performance.

## Data Availability

The original contributions presented in the study are included in the article/Supplementary material, further inquiries can be directed to the corresponding author.
